# Complete Genome Sequences of Three Streptococcus ruminantium Strains Obtained from Endocarditis Lesions of Cattle in Japan

**DOI:** 10.1128/mra.01248-21

**Published:** 2022-04-28

**Authors:** Ryohei Nomoto, Kasumi Ishida-Kuroki, Mari Tohya, Ichiro Nakagawa, Tsutomu Sekizaki

**Affiliations:** a Department of Infectious Diseases, Kobe Institute of Health, Kobe, Japan; b Antimicrobial Resistance Research Center, National Institute of Infectious Diseases, Tokyo, Japan; c Department of Microbiology, Juntendo University School of Medicine, Tokyo, Japan; d Department of Microbiology, Graduate School of Medicine, Kyoto University, Kyoto, Japan; e Graduate School of Agricultural and Life Sciences, The University of Tokyo, Tokyo, Japan; University of Maryland School of Medicine

## Abstract

Streptococcus ruminantium is a close relative of Streptococcus suis, an important zoonotic pathogen that causes various diseases in pigs and humans. Here, we report the complete genome sequences of three S. ruminantium strains isolated from bovine endocarditis in Japan.

## ANNOUNCEMENT

Streptococcus ruminantium, formerly classified as serotype 33 of the zoonotic pathogen Streptococcus suis, has recently been taxonomically reclassified as a novel streptococcal species (1). In 2018, the complete genome sequence of the type strain of S. ruminantium was published (2). However, whole-genome information on S. ruminantium is still limited, and its biological and pathological characteristics remain unclear. Here, we report the complete genome sequences of three S. ruminantium strains, GUT-183, GUT-184, and GUT-189, which were isolated from bovine endocarditis and kindly gifted to our group by the Meat Inspection Center of Sendai-City, Miyagi, Japan (1, 2).

The three S. ruminantium strains were cultured on Todd-Hewitt agar for 24 h at 37°C in 5% CO_2_. The cultures grown on the plates were subjected to lysis treatment with 50 mg/mL lysozyme, treated with 200 U/mL mutanolysin from Streptomyces globisporus ATCC 21553 (Sigma-Aldrich, USA), and incubated for 1 h at 37°C, and then the genomic DNA was extracted using the NucleoBond high-molecular-weight (HMW) DNA kit according to the manufacturer’s protocol. All genomic DNA samples were sequenced by the Taniguchi Dental Clinic-Oral Microbiome Center (Kagawa, Japan), following the standard workflow for library preparation. For short-read sequencing, genomic libraries were prepared using the MGIEasy FS PCR-free DNA library preparation set (MGI), and sequencing was performed with the DNBSEQ-G400FAST sequencer with the DNBSEQ-G400RS high-throughput rapid sequencing set (2 × 150 bp). The raw reads were quality filtered and trimmed using fastp v0.23.2 (3), with default settings. Library preparation for Oxford Nanopore Technologies (ONT) sequencing was performed using the ligation sequencing kit SQK-LSK109 (ONT, UK) without DNA fragmentation and size selection, and the libraries were sequenced using a single R9.4.1/FLO-MIN106 flow cell on a GridION X5 sequencer (ONT) with MinION software v20.10.6. Base calling was conducted using Guppy v4.2.3 in the accurate mode implemented in the MinION software. The ONT raw reads were demultiplexed, and ONT adapters were trimmed using Porechop v0.2.4 (https://github.com/rrwick/Porechop). The number of reads for each strain is listed in Table 1.

**TABLE 1:** Assembly metrics and annotated features of three Streptococcus ruminantium strains isolated from bovine endocarditis

Strain	Year of isolation	Genome size (bp)	No. of contig(s)	No. of MGI reads	No. of Nanopore reads	G+C content (%)	Total no. of CDSs^*a*^	Nanopore read *N*_50_ (bp)	GenBank accession no.	DRA accession no.
GUT-183	2001	2,175,328	1	3,918,020	149,643	39.9	1,974	20,856	AP025331	DRR332820, DRR332828
GUT-184	2003	2,115,310	1	4,349,858	131,270	40.0	1,931	12,360	AP025332	DRR332821, DRR332829
GUT-189	1993	2,081,190	1	3,884,948	141,427	40.1	1,922	14,287	AP025333	DRR332822, DRR332830

a DSs, coding DNA sequences.

Hybrid assemblies with the ONT and MGI data were performed using the Unicycler pipeline v0.4.8 (4) with default settings. Within Unicycler, the MGI reads were assembled using SPAdes v3.15.2 (5), and the resulting long-anchor contigs were assembled together with the ONT reads with an optimized version of miniasm (6) and Racon v1.4.20 (7). Pilon v1.24 (8) was used within Unicycler to iteratively polish the assembly with the MGI reads. The circularity of each contig was confirmed using the Unicycler log files. The circularized genome was rotated to the default starting gene, *dnaA*. The chromosome sequences were annotated using the DDBJ Fast Annotation and Submission Tool (DFAST) (9). Assembly metrics (genome size and number of contigs) and annotated features (numbers of coding DNA sequences, tRNAs, and rRNAs and G+C contents) are shown in Table 1.

### Data availability.

The complete genome sequences and raw sequence data for the three strains were deposited in DDBJ/EMBL/GenBank under BioProject accession number PRJDB10858. The GenBank accession numbers for the complete genome sequences are AP025331, AP025332, and AP025333. The DRA accession numbers are DRR332820, DRR332821, and DRR332822 for the DNBSEQ read data and DRR332828, DRR332829, and DRR332830 for the ONT read data.
